# Estimating the costs for the treatment of abortion complications in two public referral hospitals: a cross-sectional study in Ouagadougou, Burkina Faso

**DOI:** 10.1186/s12913-016-1822-7

**Published:** 2016-10-07

**Authors:** Patrick G. C. Ilboudo, Giulia Greco, Johanne Sundby, Gaute Torsvik

**Affiliations:** 1 Département de Santé Publique, Unité de Recherche Politiques et Systèmes de Santé, Centre MURAZ, 2054 Avenue Mamadou Konaté, 01 BP 390, Bobo-Dioulasso, Burkina Faso; 2 Department of Community Medicine, University of Oslo, Oslo, Norway; 3 London School of Hygiene and Tropical Medicine, Department of Global Health and Development, Health Economics and Systems Analysis Group, London, UK; 4 University of Bergen and Chr Michelsen Institute, Bergen, Norway

**Keywords:** Induced abortion, Complications, Treatment, Costs, Hospitals, Ouagadougou, Burkina Faso

## Abstract

**Background:**

Treatment costs of induced abortion complications can consume a substantial amount of hospital resources. This use of hospitals scarce resources to treat induced abortion complications may affect hospitals’ capacities to deliver other health care services. In spite of the importance of studying the burden of the treatment of induced abortion complications, few studies have been conducted to document the costs of treating abortion complications in Burkina Faso. Our objective was to estimate the costs of six abortion complications including incomplete abortion, hemorrhage, shock, infection/sepsis, cervix or vagina laceration, and uterus perforation treated in two public referral hospital facilities in Ouagadougou and the cost saving of providing safe abortion care services.

**Methods:**

The distribution of abortion-related complications was assessed through a review of postabortion care-registers combined with interviews with key informants in maternity wards and in hospital facilities. Two structured questionnaires were used for data collection following the perspective of the hospital. The first questionnaire collected information on the units and the unit costs of drugs and medical supplies used in the treatment of each complication. The second questionnaire gathered information on salaries and overhead expenses. All data were entered in a spreadsheet designed for studying abortion, and analyses were performed on Excel 2007.

**Results:**

Across six types of abortion complications, the mean cost per patient was USD45.86. The total cost to these two public referral hospital facilities for treating the complications of abortion was USD22,472.53 in 2010 equivalent to USD24,466.21 in 2015. Provision of safe abortion care services to women who suffered from complications of unsafe induced abortion and who received care in these public hospitals would only have cost USD2,694, giving potential savings of more than USD19,778.53 in that year.

**Conclusions:**

The treatment of the complications of abortion consumes a significant proportion (up to USD22,472.53) of the two public hospitals resources in Burkina Faso. Safe abortion care services may represent a cost beneficial alternative, as it may have saved USD19,778.53 in 2010.

**Electronic supplementary material:**

The online version of this article (doi:10.1186/s12913-016-1822-7) contains supplementary material, which is available to authorized users.

## Background

Unsafe induced abortion is widely accepted as a major health problem affecting women’s health in most developing countries and a significant cause of economic drain on the health care system. Numerous studies in various settings have shown that high proportions of gynecological admissions to hospital facilities result from complications of unsafe induced abortion [1–3]. It has been demonstrated that the provision of postabortion care to the women who had induced abortion involves huge costs and thus diminishes the health care system’s capacity to provide other needed services. Because of this, the 1994 International Conference on Population and Development Program of Action pressed countries to tackle abortion and its consequences [4]. In an effort to address the phenomenon, access to family planning services [5] and implementation of safe abortion care services have been widely recommended [6].

Several studies have highlighted the positive effects of access to family planning and safe abortion on the reduction of unwanted pregnancies [7] and maternal deaths [8, 9]. Yet, many women still resort to abortions, often in unsafe conditions, particularly in Africa [10] – where unavailability of safe abortion care services go along with mostly restrictive abortion laws [11]. Evidence has demonstrated that most complications and deaths from unsafe abortions could have been prevented [12, 13] with, among other efforts, the availability of qualified safe abortion care services. Evidence has also shown that treatment of complications related to unsafe abortion consumes significant amounts of health-system resources in Ethiopia [14], in Nigeria [15], in Colombia [16], in Uganda [17] leading to unnecessary burdens on these already stretched systems [3]. In spite of the increasing evidence of the economic consequences of abortion, [18] there have been relatively few studies that have addressed the costs of treating complications from unsafe abortions and the cost saving for providing safe abortion care services at the individual, hospital and health system levels. Particularly for Burkina Faso, while the incidence of abortion has been recognized, [1, 19] no study has examined the costs to hospitals for treating complications of unsafe abortion and the cost saving of safe abortion care services provision.

Burkina Faso, a landlocked country located in West-Africa, faces a weak health system and a high poverty level. The country has an essentially young and highly fertile population of sixteen million [20]. Contraception use is low, with large disparities between poor and rich populations [21]. Abortion is available only on specific grounds, such as the intention to save the life, physical- and/or mental health of the woman, in case of fetal impairment or in case of rape or incest [22]. Because of this, a high number of unsafe abortions has been found in women in reproductive age, resulting in a significant number of hospital admissions in the two public referral hospitals in the capital city (Ouagadougou). This study aimed at estimating the costs to these public hospital facilities for treating six types of complications of unsafe abortion, and at ascertaining the cost saving to the hospitals, had safe abortion care services been available.

## Methods

### Study type

We conducted a cross-sectional descriptive study in order to estimate the costs to two public referral hospital facilities for treating six type of post-abortion complications, including incomplete abortion, hemorrhage, shock, infection/sepsis, cervix or vagina laceration, and uterus perforation, and to ascertain the cost saving to these health facilities of the provision of safe abortion care services in 2010.

### Study sample

The two public referral hospital facilities in Ouagadougou were purposely chosen for this study. The hospital facilities included a tertiary teaching hospital and a secondary level hospital with surgical capacity. They were selected to reflect postabortion services that are routinely available, and to have a sufficient demand for such services by women with abortions. Additionally, because this study intended to make a rapid assessment of the costs incurred in treating abortion complications, these health facilities offered the best alternatives in terms of completeness of data, accessibility, and expertise in order to compute sensible estimates.

### Data sources and collection

We collected the costs data pertaining to 2010 in April 2011. In each hospital, we reviewed the manual vacuum aspiration ward registers in order to assess both the number of complications from induced abortion treated and the throughput of patients treated in each health facility in 2010. All the cases were analyzed, and classified into (1) incomplete abortions or into (2) abortion with hemorrhage, (3) shock, (4) infection/sepsis, (5) cervix or vagina laceration and (6) uterus perforation, using clinical definitions. Additionally, in each hospital facility, a gynaecologist (head of the maternity ward) or a midwife (chief of the manual vacuum aspiration ward) was interviewed in order to confirm case distribution. We also conducted face-to-face interviews with up to four key administrative personnel, including a human resources director, a financial and administrative director or the person responsible for finances, a principal accountant, and a pharmacist or person responsible for the pharmaceutical store. These face-to-face interviews were intended to collect the recurrent costs of running each hospital facility.

We used two structured questionnaires for data collection. The first questionnaire collected data on units of drugs and medical supplies used for treating each type of abortion complication (See Additional file 1). In addition, a list of prices of drugs and medical supplies used for treating cases was obtained from hospital pharmacists or from the responsible for the pharmaceutical store in each hospital. The second questionnaire gathered information on the recurrent costs of running a hospital, on the wages of health personnel and non-medical staff and on the estimated time spent in treating each type of abortion complications (See Additional file 2).

Because the perspective we adopted was the one of the facility, costs that were analyzed in this study were those paid out of the hospitals’ own budgets. Therefore, staff costs of medical and non-medical personnel whose salaries were paid by the Government were not analyzed. Similarly, capital costs such as buildings or core equipment were also excluded from the analysis as they were not acquired over hospital budgets. All the cost figures were converted from Burkina Faso CFA to US dollars, using the 2010 average yearly CFA to US dollars conversion rate [23]. In order to obtain constant dollars, we deflated the estimates using the inflation factor of the US dollar, estimated at 3.2 % [24].

### Average treatment cost

The average treatment cost of any abortion complication was obtained by adding up the estimated indirect cost, using the step-down approach, to the obtained direct cost, using the bottom-up approach.

### Step-down costing

The step-down costing approach aimed at allocating all costs for running a hospital to departments providing the final output [25] – i.e. to the maternity ward, and to patients in this ward. Treating patients in hospitals requires a combination of outputs from departments that are directly and indirectly producing care. Though they provide valuable services, departments such as kitchen, laundry, etc., are not directly involved in patient care. The costs for running these departments were therefore allocated to patient departments. Similarly, overheads costs, which are also necessary for allowing hospitals to function, were also allocated to each patient, using an allocation model. The hospital recurrent costs were allocated to maternity departments using allocation criteria reflecting actual resource use.

### Bottom-up costing

The step-down costing was complemented by bottom-up costing [25]. The aim of this approach was to capture direct treatment costs, such as drugs, medical supplies, and staff costs, which were incurred by each hospital facility in treating the complications of abortion.

### Drug and medical supplies costs

The units and types of drugs and medical supplies, including gloves for examination, gauze compresses, cotton, cotton swab, suture silk, urine collecting bags, seringes with needles, used in the treatment of each type of abortion complication were gathered through a review of selected medical files. To ensure that the information collected accurately reflected hospital practice, the data were further discussed with the gynaecologist, the person responsible for the maternity ward, or with the midwife responsible for the manual vacuum aspiration ward. Additionally, a list of the prices of the drugs and medical supplies used in treating the cases was obtained from the hospital pharmacist or from the person responsible for the pharmaceutical store in each hospital. For every complication of interest, the total cost of each drug or medical supply used in treating the case was obtained by multiplying the units of each input by the cost at which it was acquired. Total expenditure on drugs and supplies necessary for treating a single case of each abortion complication was then obtained by computing the sum total of the costs spent for each drug or supply used in such a case.

### Staff costs

In each hospital facility, a list of the medical and non-medical staff working in the department of gynaecology and obstetrics whose salaries were paid directly out of the hospital budget was established by interviewing the human resources director. The gross wage of each staff member was also obtained by interviewing the human resources director. In order to estimate the average time spent by each staff member for treating each individual case of abortion complication, the head of the maternity ward and the midwife responsible for the manual vacuum aspiration ward were interviewed. The estimated cost for each staff member was obtained by multiplying the time spent in treating each type of complication by the corresponding gross wage per minute for that staff category. The total staff cost incurred in treating each case of complication was then obtained by adding up these estimations.

### Overhead costs

Annual overheads for the year 2010 were collected from the financial statements of both health facilities. They were further discussed with the financial and administrative director in the tertiary teaching hospital, or with the person responsible for finances (or principal accountant) in the secondary level hospital, in order to identify items which were entirely paid out of the hospitals’ own finances. The overhead costs allocated to the department of gynaecology and obstetrics were calculated based on the throughput of patients in this department relative to that of the whole health facility. The estimated overhead costs attributed to the gynaecology and obstetrics department was further allocated to the maternity ward, and to each patient with an abortion. Additionally, for contractual administrative personnel working for the whole hospital, salary costs were accounted for and allocated to the maternity ward and to each case of complication treated.

### Per-patient cost and estimated total cost for treating complications

The per-patient cost of treating any case of abortion complication for each hospital facility was obtained by computing the weighted average cost of all the six abortion complications we studied. For each hospital facility, the total cost of treating complications of abortion was then obtained by multiplying the number of cases treated in 2010 by the estimated per-patient cost. The estimated total cost to these two health structures was obtained by adding up the estimated costs to each hospital.

### Estimated cost saving to these two public hospitals if safe abortion care services were available in 2010

To estimate the potential saving of safe abortion care services provision for the two public hospital facilities in 2010, we used additional data from a published costing study. Under a scenario that promoted the availability and accessibility of safe abortion care services, the authors estimated that safe abortion provision would only have cost USD6 [26]. We multiplied the estimated number of complications treated in the two referral hospitals in 2010 by USD6 to obtain the total costs to these hospital facilities had women had access to safe abortion care services. Finally, we subtracted the resulting amount from the estimated total cost for treating the complications in order to ascertain the saving that would have been obtained in that year.

### Sensitivity analysis

It is widely admitted that studies investigating abortion, particularly induced abortion, are subject to numerous limitations. Among these limitations, underreporting of cases has been acknowledged as a major source of bias which affects estimates. Underreporting may be caused by misclassification of cases, particularly induced abortions into spontaneous abortions, [27, 28] even at hospital level. This study may have been affected by misclassification of cases making it necessary to conduct a sensitivity analysis. This sensitivity analysis aimed at anticipating the lack of determinism in the parameters used to estimate the total cost of treating abortion complications. In the absence of recommendations in choosing sensitivity values for abortion costing studies, previous studies which have estimated the cost of treating abortion-related complications have used various sensitivity values [14, 16, 17]. Because of this, minimum and maximum values used to test for the sensitivity of the results were set to 25 %, lesser or greater than the central estimates [16]. These minimum and maximum values are interpreted as the amount by which the total cost of treating the complications of abortion and the potential cost saving could be, respectively, decrease or increased given changes in cost parameters. Therefore, sensitivity tests of the estimated total cost of treating the complications of abortion were performed on all parameters, including: number of treated cases, unit cost of drugs and supplies, staff cost, and overheads. Additionally, we also tested the changes in the estimated saving if safe abortion care services were available in 2010, relative to the same parameters. All sensitivity tests were performed using a one-way sensitivity model built on Excel 2010.

## Results

### Estimated distribution of abortion complications in the studied hospitals

Incomplete abortion was the most common abortion complication treated in any of these public referral hospital facilities in Ouagadougou, accounting for 40 % of the cases (Table 1). Hemorrhage and shock were the second and third leading causes of admissions in these hospitals, accounting respectively for 32 and 15 % of the cases. Similar to what was found for the combined facilities, hemorrhage and shock appeared to be the second and third leading complications treated in the tertiary teaching hospital. At the district level hospital however, we found that infection/sepsis and hemorrhage appeared to share a place as the second leading cause of admissions, each accounting for 25 % of all cases. Admissions for cervix or vagina laceration and uterus perforation were rare, together accounting for only 4 % of all treated cases. Uterus perforation appeared to be treated only in the tertiary level teaching hospital.

**Table 1:** Distribution of the number of abortion complications treated in the studied hospitals

	Secondary hospital		Tertiary hospital		Both hospitals	
	n	%	n	%	n	%
Incomplete abortion	13	41	167	40	180	40
Hemorrhage	8	25	134	32	142	32
Shock	2	6	67	16	69	15
Infection/sepsis	8	25	33	8	41	9
Cervix or vagina laceration	1	3	8	2	9	2
Uterus perforation	0	0	8	2	8	2
Total	32	100	417	100	449	100

### Average treatment cost per type of complication

The result indicated that treatment costs of abortion complications in both referral hospital ranged from USD23.71 for an incomplete abortion to USD85.08 for a case of infection/sepsis (Table 2). Treatments of uterus perforation and infection/sepsis appeared to be the most costly services to public referral hospitals, while incomplete abortion and hemorrhage were the least expensive services, at USD23.71 and USD26.30 per case, respectively. We also found that uterus perforation and infection/sepsis were the most expensive services at the tertiary level teaching hospital, at a cost of USD73.76 and USD94.39, respectively. We were surprised to find, at the secondary level teaching hospital, that treatment of a case of shock was less expensive than treating hemorrhage. Treatments of cervix or vagina laceration and of infection/sepsis were the most expensive procedures at the district hospital level. In all hospitals, treatment of abortion-related infection or sepsis was the most costly procedure. Additionally, whatever the complication, estimated treatment costs were higher in the tertiary level hospital than in the secondary level hospital.

**Table 2:** Average treatment cost per type of complication (constant 2010 USD)

	Secondary hospital		Tertiary hospital		Both hospitals	
	Cost	%	Cost	%	Cost	%
Incomplete abortion						
Direct costs	14.10	65.98	13.62	52.32	13.86	58.47
Drugs and supplies	13.20	61.78	13.20	50.72	13.20	55.69
Salaries (opportunity costs)	0.90	4.19	0.41	1.59	0.66	2.78
Indirect costs	7.27	34.02	12.41	47.68	9.85	41.53
Overheads	7.27	34.02	12.41	47.68	9.85	41.53
Total	21.37	100.00	26.03	100.00	23.71	100.00
Infection / sepsis						
Direct costs	46.67	61.61	44.73	47.39	45.70	53.72
Drugs and supplies	43.07	56.85	43.07	45.63	43.07	50.63
Salaries (opportunity costs)	3.60	4.76	1.66	1.76	2.63	3.09
Indirect costs	29.09	38.39	49.65	52.61	39.37	46.28
Overheads	29.09	38.39	49.65	52.61	39.37	46.28
Total	75.76	100.00	94.39	100.00	85.08	100.00
Hemorrhage						
Direct costs	17.21	66.00	19.24	72.57	18.23	69.31
Drugs and supplies	16.92	64.88	18.34	69.19	17.64	67.05
Salaries (opportunity costs)	0.29	1.12	0.90	3.38	0.59	2.26
Indirect costs	8.87	34.00	7.27	27.43	8.07	30.69
Overheads	8.87	34.00	7.27	27.43	8.07	30.69
Total	26.08	100.00	26.51	100.00	26.30	100.00
Shock						
Direct costs	15.99	64.34	27.93	62.70	21.97	63.30
Drugs and supplies	15.70	63.16	25.88	58.09	20.80	59.90
Salaries (opportunity costs)	0.29	1.18	2.06	4.61	1.18	3.40
Indirect costs	8.87	35.66	16.62	37.30	12.74	36.70
Overheads	8.87	35.66	16.62	37.30	12.74	36.70
Total	24.86	100.00	44.55	100.00	34.72	100.00
Cervix or vagina laceration / perforation						
Direct costs	24.18	73.17	42.73	72.00	33.46	72.42
Drugs and supplies	23.89	72.29	41.70	70.27	32.80	70.99
Salaries (opportunity costs)	0.29	0.88	1.03	1.73	0.66	1.43
Indirect costs	8.87	26.83	16.62	28.00	12.74	27.58
Overheads	8.87	26.83	16.62	28.00	12.74	27.58
Total	33.05	100.00	59.35	100.00	46.20	100.00
Uterus laceration / perforation						
Direct costs	-	-	24.11	32.68	24.11	32.68
Drugs and supplies	-	-	22.45	30.43	22.45	30.43
Salaries (opportunity costs)	-	-	1.66	2.25	1.66	2.25
Indirect costs	-	-	49.65	67.32	49.65	67.32
Overheads	-	-	49.65	67.32	49.65	67.32
Total	-	-	73.76	100.00	73.76	100.00

In both referral hospital facilities direct costs of treatments accounted for more than 53 % of the total cost, except for uterus perforation, for which direct costs stood at around 33 %. Furthermore, direct costs of treatment accounted for more than 61 % of the total cost of any complication at the district level hospital, and more than 47 % at the tertiary teaching hospital. Salaries accounted only marginally for the total cost of any type of abortion complication. However, they seemed to be higher in the district hospital, compared to the tertiary teaching hospital.

### Per-patient cost

The average per-patient cost of treating any complication of abortion was USUSD45.86 (Table 3). This cost was higher in the tertiary teaching hospital compared to the secondary level hospital: USD51.09 versus USD36.50, respectively. Direct costs accounted for a minimum of 56 % of the total cost for both referral hospitals in Ouagadougou. However, they represented a much higher percentage of the hospital facility’s resources in the secondary level hospital compared to the tertiary level hospital: 63.63 % vs. 51.70 %. Opportunity costs (salaries) were slightly higher in the secondary hospital compared to the tertiary level hospital.

**Table 3:** Per-patient cost for treating any complication (constant 2010 USD)

	Secondary hospital		Tertiary hospital		Both hospitals	
	Cost	%	Cost	%	Cost	%
Direct costs	23.22	63.63	26.41	51.70	25.92	56.52
Drugs and supplies	21.86	59.89	25.35	49.62	24.58	53.59
Salaries (opportunity cost)	1.37	3.74	1.06	2.08	1.34	2.93
Indirect costs	13.28	36.37	24.68	48.30	19.94	43.48
Overheads	13.28	36.37	24.68	48.30	19.94	43.48
Total cost	36.50	100.00	51.09	100.00	45.86	100.00

### Estimated total cost for treating all complications

The total annual cost to the two public hospital facilities for treating complications of abortion rouse to USD22,472.53 in 2010 USD, equivalent to 2015 USD24,466.21 (more than CFA12 millions) (Table 4). Indirect costs (i.e. overhead costs) invested in treating complications of abortion were higher in the tertiary hospital compared to the secondary level hospital; the reverse was observed for direct costs. For both hospital facilities, drugs and supplies appeared to be the main sources of direct costs, accounting for a minimum of 50 % of all direct costs. This proportion was slightly higher in the secondary hospital compared to that observed at the tertiary hospital in Ouagadougou.

**Table 4:** Estimated total cost to the two referral hospitals for treating abortion complications (constant 2010 USD)

	Secondary hospital		Tertiary hospital		Total hospital costs	
	Cost	%	Cost	%	Cost	%
Direct costs	743.04	63.62	11012.97	51.69	11756.01	52.31
Drugs and supplies	699.52	59.89	10570.95	49.62	11270.47	50.15
Salaries (opportunity costs)	43.84	3.75	442.02	2.07	485.86	2.16
Indirect costs	424.96	36.38	10291.56	48.31	10716.52	47.69
Overheads	424.96	36.38	10291.56	48.31	10716.52	47.69
Total cost	1168.00	100.00	21304.53	100.00	22472.53	100.00

### Estimated cost saving if safe abortion care services were available in 2010

Our calculations showed that the provision of safe abortion care services would only have cost USD2,694 for both referral hospital facilities. This amount, broadly equivalent to just CFA 1,3 million, would have represented only 12 % of the total cost spent in treating the complications of unsafe abortions in the two health facilities (Table 5). An estimated USD19,778.53, equivalent to more than CFA9.7 million, would therefore have been saved in 2010 if safe abortion care services were available.

**Table 5:** Estimated saving (constant 2010 USD) if safe abortion care services were available in 2010

	Estimated cost (USD)	Estimated cost (CFA)
Restricted abortion services scenario		
Cost of treating complications	22,472.53	11.085.708,00
Liberal abortion services scenario		
Cost of safe abortion procedures	2,694.00	1.328.951,25
Estimated total potential saving	19,778.53	9.756.756,57

### Sensitivity analyses

Tables 6 and 7 show the results of the one way sensitivity analyses of the total estimated costs of complication treatments and cost saving of implementing safe abortions services relative to changes in: the number of complications treated in each hospital facility; the estimated unit cost of drugs and supplies used; the staff cost; the estimated overhead costs; and finally, in the estimated unit cost of qualified abortion service provision. The findings suggested that the estimated total cost for treating the complications of abortion was mostly sensitive to the numbers of complications treated in both hospital facilities (Table 6). However, the variation in cost estimates was larger in the tertiary level hospital compared to the secondary hospital.

**Table 6:** Sensitivity analysis of the total cost (in USD) for treating complications of abortion in the two hospitals in 2010

	Minimum	Medium	Maximum
Secondary hospital			
Number of cases	22,180.77	22,472.53	22,764.93
Drugs and supplies	22,298.13	22,472.53	22,647.89
Salaries (opportunity costs)	22,461.97	22,472.53	22,483.73
Overheads	22,366.61	22,472.53	22,579.09
Tertiary hospital			
Number of cases	17,146.72	22,472.53	27,798.98
Drugs and supplies	19,829.07	22,472.53	25,116.63
Salaries (opportunity costs)	22,364.43	22,472.53	22,585.44
Overheads	19,899.96	22,472.53	25,045.74

**Table 7:** Sensitivity analysis of the estimated saving of safe abortion care services provision in 2010 in (USUSD)

	Minimum	Medium	Maximum
Number of cases in the secondary hospital	19,730.85	19,778.53	19,826.85
Number of cases in the tertiary hospital	19,153.35	19,778.53	20,404.35
Estimated qualified care cost	19,105.35	19,778.53	20,452.35

Unsurprisingly, the estimated saving of safe abortion care services provision was also sensitive to the same parameters i.e. to the numbers of complications treated in the hospital facilities, but more sensitive to the estimated cost of the abortion care service offered under a liberal scenario which permits it (Table 7).

## Discussion

In this paper, we have investigated the costs to two public referral hospital facilities in Ouagadougou for treating the most common complications of abortion and have examined the potential cost saving to these hospitals had safe abortion care services been available in 2010. Despite the restrictive law prevailing in Burkina Faso, the findings showed a non-negligible number of women being treated for abortion-related complications, particularly in the tertiary hospital. As the tertiary hospital is a national hospital to which all other hospital facilities refer cases, we were not surprised to find a higher throughput of patients treated for complications of abortion there. However, we believe that methodological challenges related to the controversy surrounding abortion mean that the numbers we have presented likely underestimate the true magnitude of unsafe abortion complications treated in these hospital facilities [1, 19]. Misclassification of abortion cases is one of the most important methodological challenges. We believe some women who have had induced abortions may have reported having had spontaneous abortions. This may have affected the number and distribution of abortion related complications. Additionally, the distribution of the abortion-related complications that we found in the selected hospitals may not reflect the true distribution of abortion-related complications. We believe that some of the complications treated in these hospitals may not have been declared as resulting from induced procedures or registered in hospitals records. This may have been exacerbated by taboo, fear, and shame, [19] which may have led some women to request hospital care clandestinely, and only at night.

The results also indicate that the average treatment cost of an abortion complication amounted to USD45,86, rising beyond the per capita expenditure on health care in Burkina Faso, which has been estimated to USD39 [29]. This gives a total estimated cost of USD22,472.53 for these two hospital facilities, with the tertiary level hospital bearing most of the economic consequences of treating complications of abortions: USD21,304.53 vs. USD1,168.00. Furthermore, we found that the availability of safe abortion care services would have saved USD19,778.53 for these two hospital facilities in 2010. In line with previous studies, this reinforces the argument that safe abortion care services represent a cost effective strategy [30].

We also found that the indirect cost for treating a complication of abortion was relatively high, rising up to almost 38 % of the total cost for both referral hospital facilities in Ouagadougou. This percentage was higher in the tertiary level hospital compared to the secondary hospital. This may suggest that a reduction of the estimated cost invested in treating any abortion complication is achievable, particularly in the tertiary level hospital, if the throughput of women requesting treatment for abortion complications in hospitals increases. We believe this to be the case because indirect costs are fixed, that is, they are less likely to vary over time. Moreover, with multiple requests for resources’ use, some more urgent than others, it’s crucial to choose where to invest basing on accurate data and thorough analysis of costs. This cost analysis may therefore help hospital administrators invest their scarce resources strategically and draft an effective budget for projected expenditures. The estimated total costs for treating the complications of abortion and the potential saving of the provision of safe abortion care services both varied relative to the number of cases treated in the hospitals. This, too, has some implications. Again in line with the literature, [31] it may suggest that more effort should be devoted to producing accurate estimates of the incidence and numbers of complications of abortion treated in hospitals.

This study has some limitations which we must take into account. In estimating the costs to these hospital facilities for treating the complications of abortion, we purposely selected two hospitals rather than sampling them randomly. Because of this, the estimates of costs we have presented here may not be generalizable to the whole country. It has not been possible to present national estimates of both the treatment of abortion complications and the potential saving to the entire health system if safe abortions services were available in 2010. Future studies should therefore look into the costs to the entire health care system for treating the complications of unsafe abortions. Moreover, in estimating the potential cost saving if safe abortion care services were available in 2010, we adopted the naïve scenario that all women who would benefit from safe abortion care services would be spared all possible complications. In reality, even if safe abortion care services were available, it is likely that some women would still experience complications, which would have consumed a much higher share of hospital resources than what is accounted for in this scenario. Therefore, the estimate of the cost saving we presented in this study must have overestimated the true benefit to these hospital facilities if safe abortion care services were available. In addition, in estimating the potential cost saving if safe abortion care services were available in 2010, we assumed that all the women who have had unsafe abortion would have accessed safe abortion care services. Again, this scenario is unrealistic. Despite the availability of safe abortion care services in some countries, numerous evidences have shown that some women still experience barriers for accessing these services. Our estimate of the potential saving to these two hospital facilities may have therefore been overestimated because we did not take this parameter into account. Finally, the USD6 figure is an estimate of the direct costs borne by an hospital facility for the provision of safe abortion care services under a liberalized scenario cost [26]. Therefore, the use of the USD6 figure may lead to an underestimation of the true costs to the hospital facility for providing safe abortion care service given that the provision of care encompasses a combination of direct and indirect costs. This in turn, may have resulted in an overestimation of the potential cost saving to the hospital facility had safe abortion care services been available.

Although we have presented the value of this information in terms of strategies for reducing costs, the collection and analysis of cost data also has wider implications that concern the hospitals: Though they were not acquired through the budgets of the hospital facilities, resources such as buildings and core equipment will depreciate, and the treatment of abortion complications contributes to this depreciation. In analyzing the cost from the perspective of the hospital, we failed to capture the cost incurred due to depreciation, and therefore underestimated the real costs to the health system for providing postabortion care. Finally, we only reviewed manual vacuum aspiration cases and not the other treatment of abortion complications. This also could have also contributed to underestimating the incurred costs of treating complications from induced abortion. Despite the limitations we mentioned, this study has some strengths. First, it is the first study attempting to estimate the costs for the treatment of abortion complications to referral hospital facilities in Burkina Faso. The vast majority of the studies of abortion issues in Burkina Faso have focused on the epidemiological, clinical, or anthropological aspects of abortion and postabortion care rather than on economic aspects. Second, because the hospital facilities in which the study was conducted cover a huge population with limited budgets, this study may be valuable as a starting point for minimizing costs. Finally, it could serve as a basis for designing a larger and more representative research project on the costs to the entire health system for treating the complications of abortions.

## Conclusions

This study undoubtedly provides an important piece of information on the burden of unsafe abortion to hospital facilities in Ouagadougou. It demonstrates that the treatment of abortion complications consumes a substantial amount of referral hospital facilities’ resources in Ouagadougou. It also highlights the fact that implementation of safe abortion care services may clearly reduce the burden on hospitals of treating complications of abortion. Therefore, there is a need for providing safe abortion care services that are affordable in all facilities of the country at large. This provision of safe abortion care services by hospital facilities may be organized as a routine outpatient day-service with relatively low hospital costs, whereas treatments of abortion complications are non-scheduled events, and are therefore more costly.

## Additional files

Additional file 1: This questionnaire was designed to collect direct costs of treating a patient. (PDF 566 kb)
Additional file 2: This questionnaire was designed to collect indirect costs borne by hospital facilities in treating patients. (PDF 327 kb)

## Abbreviations

CFA: Currency of the African Financial Community
USD: United States Dollars
