# Supplementary material for: Estimating the costs for the treatment of abortion complications in two public referral hospitals: a cross-sectional study in Ouagadougou, Burkina Faso
Source: BMC Health Serv Res. 2016 Oct 7;16:559. doi: 10.1186/s12913-016-1822-7 (PMC5055714; doi:10.1186/s12913-016-1822-7)
Supplement: Additional file 1: — This questionnaire was designed to collect direct costs of treating a patient. (PDF 566 kb) [file 12913_2016_1822_MOESM1_ESM.pdf]

# <u>COST OF ABORTIONS</u>

## Drugs, materials and consumables used in treating abortion complications (Unofficial translation)

This questionnaire comprise 6 parts :

Part A : Patients suffering from incomplete abortion
Part B : Patients suffering from sepsis/infection
Part C : Patients suffering from haemorrhage
Part D : Patients suffering from shock
Part E : Patients suffering from of vagina or cervix laceration/perforation
Partie F : Patients suffering from uterus laceration/perforation

| PART A : Patients suffering from incomplete abortion | | | | | | |
|---|---|---|---|---|---|---|
| N° | Name of input | Percent of **outpatients** suffering from incomplete abortion who received this input<br><br>0%=no women<br>100%=all women | Percent of **inpatients** suffering from incomplete abortion who received this input<br><br>0%=no women<br>100%=all women | Unit<br>(**mg**=milligram,<br>**mcg**=microgram,<br>**ml**=milliliter<br>**ui=**international unit<br>**unit**=unit, piece… | Average number of basic units given per **outpatient** who receives this input | Average number of basic units given per **inpatient** who receives this input |
| (1) | (2) | (3) | (4) | (5) | (6) | (7) |
| A1 | Percent of women suffering from incomplete abortion who are treated as outpatients<br>0%=none treated as outpatients<br>100%=All treated as outpatients | % | % | | | |
| | 1. Antibiotics | | | | | |
| A12 | Amoxicillin 500mg | | | mg | | |
| A13 | Ampicillin, 1gram, powder for inj | | | mg | | |
| A14 | Ampicillin, tablets, 500mg | | | mg | | |
| A15 | Ceftriaxon, 1g, , powder for inj | | | mg | | |
| A16 | Chloramphenicol, 1g, for injection | | | | | |
| A17 | Ciprofloxacin, 500mg | | | mg | | |
| A18 | Doxycycline 100mg | | | mg | | |
| A19 | Gentamycin 80mg/2ml for injection | | | mg | | |
| A110 | Metronidazole injection 5mg/ml, 100ml | | | mg | | |
| A111 | Metronidazole 500mg vaginal | | | mg | | |
| | 2. Analgesics | | | | | |
| A21 | Acetylsalicylic acid, 100mg | | | mg | | |
| A22 | Diazepam, 10mg/2ml, for injection (valium) | | | mg | | |
| A23 | Diclofenac sodium, 75mg/3ml, for injection | | | mg | | |
| A24 | Diclofenac 100mg, rectal suppository | | | mg | | |
| A25 | Butilhisocin, 20g/ml, 1ml, for injection | | | mg | | |

| | | | | | | |
|---|---|---|---|---|---|---|
| **PART A : Patients suffering from incomplete abortion** | | | | | | |
| A26 | Ibuprofen 400mg, film coated | | | mg | | |
| A27 | Metronidazole 500mg (metamizole) | | | mg | | |
| A28 | Paracetamol, tablets, 500mg | | | mg | | |
| A29 | Pethidine hydrochloride 50mg/ml, 1ml, for injection | | | mg | | |
| | | | | | | |
| | 3. Intravenous fluid replacement | | | | | |
| A31 | Ringer lactate, 1000ml, pvc bag | | | ml | | |
| A32 | Glucose isotonic 5%, intravenous solution 500ml, bag/bottle | | | ml | | |
| A33 | Glucose 5% + sodium chloride 0,9% IV, solution 500ml bag/bottle | | | ml | | |
| A34 | Sodium chloride 0,9%, 1000ml, bag | | | ml | | |
| | | | | | | |
| | 4. Control of bleeding | | | | | |
| A41 | Ergotamine 1mg+caffeine 100mg | | | mg | | |
| A42 | Ergometrine maleate 0,2 mg | | | mg | | |
| A43 | Ocytocin10ui/ml, 1ml, for injection | | | ui | | |
| A44 | Misoprostol 20mg | | | mg | | |
| A45 | Dypron, dipyrone 250mg/ml, 2ml, injection | | | mg | | |
| | | | | | | |
| | 5. Lab tests | | | | | |
| A51 | Glycemy, PK50 | | | unit | | |
| A52 | GS/Rh 200tests, 10 ml | | | ml | | |
| A53 | Blood lancet, disposable | | | unit | | |
| A54 | Blood taking set with needle, disposable | | | unit | | |
| A55 | Ethanol 70% denaturated, 1 liter | | | ml | | |
| A56 | Gauze compresse 5*5cm (per 5 pieces) | | | unit | | |
| A57 | Hemoglobin test | | | unit | | |
| A58 | Hepapitis C rapid test | | | unit | | |

| | | | | | | |
|---|---|---|---|---|---|---|
| **PARTA : Patients suffering from incomplete abortion** | | | | | | |
| A59 | Seringe 5ml with bypacked needle 21G | | | unit | | |
| A510 | Ultrasound procedure | | | unit | | |
| A511 | Urine collecting bag 2000ml, with tap and valve | | | unit | | |
| | | | | | | |
| | 6. Operating theater supplies | | | | | |
| A61 | Seringe 10ml, bypacked needle 21G | | | ml | | |
| A62 | Seringe 60ml+catheter | | | ml | | |
| A63 | Gloves examination, latex, large, disposable | | | unit | | |
| A64 | Cotton hydrophile, 500g | | | mg | | |
| A65 | Gauze compresse 5*5cm, 12ply, non sterile | | | unit | | |
| A66 | Ehanol 70%, denaturated, 1liter | | | ml | | |
| A67 | Cetrimide 15% + chlorhexidine gluconate 1,5%, 1liter | | | ml | | |
| A68 | Misoprostol, 200mg | | | mg | | |
| A69 | Povidone iodine solution, 0.1, 200ml | | | ml | | |
| A610 | Sanitary pad | | | unit | | |
| | | | | | | |
| | 7. Anesthesics/sedatives | | | | | |
| A71 | Atropine 1mg/ml, 1ml for injection | | | mg | | |
| A72 | Diazepam, 10mg/2ml, for injection | | | mg | | |

| PART B : Patients suffering from sepsis/infection | | | | | | |
|---|---|---|---|---|---|---|
| N° | Name of input | Percent of **outpatients** suffering from sepsis/infection who received this input<br><br>0%=no women<br>100%=all women | Percent of **inpatients** suffering from sepsis/infection who received this input<br><br>0%=no women<br>100%=all women | Unit (**mg**=milligram, **mcg**=microgram, **ml**=milliliter **ui=**international unit **unit**=unit, piece… | Average number of basic units given per **outpatient** who receives this input | Average number of basic units given per **inpatient** who receives this input |
| B1 | Percent of women suffering from sepsis/infection who are treated as outpatients<br>0%=none treated as outpatients<br>100%=All treated as outpatients | % | % | | | |
| | 1. Antibiotics | | | | | |
| B12 | Amoxicillin 500mg | | | mg | | |
| B13 | Ampicillin, 1gram, powder for inj | | | mg | | |
| B14 | Ampicillin, tablets, 500mg | | | mg | | |
| B15 | Ceftriaxon, 1g, , powder for inj | | | mg | | |
| B16 | Chloramphenicol, 1g, for injection | | | mg | | |
| B17 | Ciprofloxacin, 500mg | | | mg | | |
| B18 | Doxycycline 100mg | | | mg | | |
| B19 | Gentamycin 80mg/2ml for injection | | | mg | | |
| B110 | Metronidazole injection 5mg/ml, 100ml | | | mg | | |
| B111 | Metronidazole 500mg vaginal | | | mg | | |
| | 2. Intravenous fluid replacement | | | | | |
| B21 | Ringer lactate, 1000ml, pvc bag | | | ml | | |
| B22 | Glucose isotonic 5%, intravenous solution 500ml, bag/bottle | | | ml | | |
| B23 | Glucose 5% + sodium chloride 0,9% IV, solution 500ml bag/bottle | | | ml | | |
| B24 | Sodium chloride 0,9%, 1000ml, bag | | | ml | | |
| | 3. Management of respiration | | | | | |
| B31 | Oxygen, 100000ml | | | ml | | |

| PART B : Patients suffering from sepsis/infection | | | | | | |
|---|---|---|---|---|---|---|
| | 4. Lab tests | | | | | |
| B41 | Glycemy, PK50 | | | unit | | |
| B42 | GS/Rh 200tests, 10 ml | | | ml | | |
| B43 | Blood lancet, disposable | | | unit | | |
| B44 | Blood taking set with needle, disposable | | | unit | | |
| B45 | Ethanol 70% denaturated, 1 liter | | | ml | | |
| B46 | Gauze compresse 5*5cm (per 5 pieces) | | | unit | | |
| B47 | Hemoglobin test | | | unit | | |
| B48 | Hepapitis C rapid test | | | unit | | |
| B49 | Seringe 5ml with bypacked needle 21G | | | unit | | |
| B410 | Ultrasound procedure | | | unit | | |
| B411 | Urine collecting bag 2000ml, with tap and valve | | | unit | | |
| | 5. Others | | | | | |
| B51 | Tetanus vaccine 20 doses (unit) | | | unit | | |
| B52 | Ferrous sulphate 200mg + folic acid 0,25mg | | | mg | | |
| B53 | Dypron, dipyrone 250mg/ml, 2ml, injection | | | unit | | |
| | | | | | | |
| | 6. Anesthesics/sedatives | | | | | |
| B61 | Atropine 1mg/ml, 1ml for injection | | | mg | | |
| B62 | Diazepam, 10mg/2ml, for injection | | | mg | | |
| B63 | Fentanyl 0,1mg/2ml, for injection | | | mg | | |
| B64 | Halothane 250ml | | | ml | | |
| B65 | Ketamine 50mg/ml, 10ml for injection | | | mg | | |
| B66 | Lidocaïne HCl 1%, 20ml for injection | | | ml | | |
| B67 | Lidocaïne hydrochloride+ epinephrine | | | ml | | |
| B68 | Lidocaïne HCl 5%+dextrose 7,5%, 2ml for injection | | | ml | | |
| B69 | Thiopental sodium 1g, powder for injection | | | mg | | |
| B610 | Suxamethonium chloride 100mg/2ml for injection | | | mg | | |

| PART C : Patients suffering from haemorrhage | | | | | | |
|---|---|---|---|---|---|---|
| N° | Name of input | Percent of **outpatients** suffering from haemorrhage who received this input<br><br>0%=no women<br>100%=all women | Percent of **inpatients** suffering from haemorrhage who received this input<br><br>0%=no women<br>100%=all women | Unit<br>(**mg**=milligram,<br>**mcg**=microgram,<br>**ml**=milliliter<br>**ui=**international unit<br>**unit**=unit, piece… | Average number of basic units given per **outpatient** who receives this input | Average number of basic units given per **inpatient** who receives this input |
| C1 | Percent of women suffering from haemorrhage who are treated as outpatients<br>0%=none treated as outpatients<br>100%=All treated as outpatients | % | % | | | |
| | 1. Respiration management | | | | | |
| C1 | Oxygen, 100000ml | | | ml | | |
| | | | | | | |
| | 2. Control bleeding | | | | | |
| C21 | Ergotamine 1mg+caffeine 100mg | | | mg | | |
| C22 | Ergometrine maleate 0,2 mg | | | mg | | |
| C23 | Ocytocin10ui/ml, 1ml, for injection | | | ui | | |
| C24 | Misoprostol 20mg | | | mg | | |
| C25 | Dypron, dipyrone 250mg/ml, 2ml, injection | | | mg | | |
| | | | | | | |
| | 3. Analgesics | | | | | |
| C31 | Acetylsalicylic acid, 100mg | | | mg | | |
| C32 | Diazepam, 10mg/2ml, for injection (valium) | | | mg | | |
| C33 | Diclofenac sodium, 75mg/3ml, for injection | | | mg | | |
| C34 | Diclofenac 100mg, rectal suppository | | | mg | | |
| C35 | Butilhisocin, 20g/ml, 1ml, for injection | | | mg | | |
| C36 | Ibuprofen 400mg, film coated | | | mg | | |
| C37 | Metronidazole 500mg (metamizole) | | | mg | | |
| C38 | Paracetamol, tablets, 500mg | | | mg | | |
| C39 | Pethidine hydrochloride 50mg/ml, 1ml, for injection | | | mg | | |

| PART C : Patients suffering from haemorrhage | | | | | | |
|---|---|---|---|---|---|---|
| | 4. Intravenous fluid replacement | | | | | |
| C41 | Ringer lactate, 1000ml, pvc bag | | | ml | | |
| C42 | Glucose isotonic 5%, intravenous solution 500ml, bag/bottle | | | ml | | |
| C43 | Glucose 5% + sodium chloride 0,9% IV, solution 500ml bag/bottle | | | ml | | |
| C44 | Sodium chloride 0,9%, 1000ml, bag | | | ml | | |
| C45 | Polygeline 4%, 500ml (gelofusine, hemacel) | | | ml | | |
| C46 | Dextrose 2,5%+NaCl 0,45%, 1000ml bag | | | ml | | |
| | | | | ml | | |
| | 5. Blood transfusion | | | | | |
| C51 | Blood, unit, 500ml | | | unit | | |
| C52 | Ferrous sulphate 200mg + folic acid 0,25mg | | | mg | | |
| | | | | | | |
| | 6. Lab tests | | | | | |
| C61 | Glycemy, PK50 | | | unit | | |
| C62 | GS/Rh 200tests, 10 ml | | | ml | | |
| C63 | Blood lancet, disposable | | | unit | | |
| C64 | Blood taking set with needle, disposable | | | unit | | |
| C65 | Ethanol 70% denaturated, 1 liter | | | ml | | |
| C66 | Gauze compresse 5*5cm (per 5 pieces) | | | unit | | |
| C67 | Hemoglobin test | | | unit | | |
| C68 | Hepapitis C rapid test | | | unit | | |
| C69 | Seringe 5ml with bypacked needle 21G | | | unit | | |
| C610 | Ultrasound procedure | | | unit | | |
| C611 | Urine collecting bag 2000ml, with tap and valve | | | unit | | |
| | | | | | | |
| | | | | | | |

| PART C : Patients suffering from haemorrhage | | | | | | |
|---|---|---|---|---|---|---|
| | 7. Anesthesics/sedatives | | | | | |
| C71 | Atropine 1mg/ml, 1ml for injection | | | mg | | |
| C72 | Diazepam, 10mg/2ml, for injection | | | mg | | |
| C73 | Fentanyl 0,1mg/2ml, for injection | | | mg | | |
| C74 | Halothane 250ml | | | ml | | |
| C75 | Ketamine 50mg/ml, 10ml for injection | | | mg | | |
| C76 | Lidocaïne HCl 1%, 20ml for injection | | | ml | | |
| C77 | Lidocaïne hydrochloride+ epinephrine | | | ml | | |
| C78 | Lidocaïne HCl 5%+dextrose 7,5%, 2ml for injection | | | ml | | |
| C79 | Thiopental sodium 1g, powder for injection | | | mg | | |
| C710 | Suxamethonium chloride 100mg/2ml for injection | | | mg | | |
| | | | | | | |
| | | | | | | |

| PART D : Patients suffering from shock | | | | | | |
|---|---|---|---|---|---|---|
| N° | Name of input | Percent of **outpatients** suffering from shock who received this input<br><br>0%=no women<br>100%=all women | Percent of **inpatients** suffering from shock who received this input<br><br>0%=no women<br>100%=all women | Unit (**mg**=milligram, **mcg**=microgram, **ml**=milliliter **ui=**international unit **unit**=unit, piece… | Average number of basic units given per **outpatient** who receives this input | Average number of basic units given per **inpatient** who receives this input |
| D1 | Percent of women suffering from shock who are treated as outpatients<br>0%=none treated as outpatients<br>100%=All treated as outpatients | % | % | | | |
| | 1. Management of respiration | | | | | |
| D11 | Oxygen, 100000ml | | | ml | | |
| | | | | | | |
| | 2. Control of bleeding | | | | | |
| D21 | Ergotamine 1mg+caffeine 100mg | | | mg | | |
| D22 | Ergometrine maleate 0,2 mg | | | mg | | |
| D23 | Ocytocin10ui/ml, 1ml, for injection | | | ui | | |
| D24 | Misoprostol 20mg | | | mg | | |
| D25 | Dypron, dipyrone 250mg/ml, 2ml, injection | | | mg | | |
| | | | | | | |
| | 3. Analgesics | | | | | |
| D31 | Acetylsalicylic acid, 100mg | | | mg | | |
| D32 | Diazepam, 10mg/2ml, for injection (valium) | | | mg | | |
| D33 | Diclofenac sodium, 75mg/3ml, for injection | | | mg | | |
| D34 | Diclofenac 100mg, rectal suppository | | | mg | | |
| D35 | Butilhisocin, 20g/ml, 1ml, for injection | | | mg | | |
| D36 | Ibuprofen 400mg, film coated | | | mg | | |
| D37 | Metronidazole 500mg (metamizole) | | | mg | | |
| D38 | Paracetamol, tablets, 500mg | | | mg | | |
| D39 | Pethidine hydrochloride 50mg/ml, 1ml, for inj | | | mg | | |

| **PART D : Patients suffering from shock** | | | | | | |
|---|---|---|---|---|---|---|
| | 4. Intravenous fluid replacement | | | | | |
| D41 | Ringer lactate, 1000ml, pvc bag | | | ml | | |
| D42 | Glucose isotonic 5%, intravenous solution 500ml, bag/bottle | | | ml | | |
| D43 | Glucose 5% + sodium chloride 0,9% IV, solution 500ml bag/bottle | | | ml | | |
| D44 | Sodium chloride 0,9%, 1000ml, bag | | | ml | | |
| D45 | Polygeline 4%, 500ml (gelofusine, hemacel) | | | ml | | |
| D46 | Dextrose 2,5%+NaCl 0,45%, 1000ml bag | | | ml | | |
| | | | | ml | | |
| | 5. Blood transfusion | | | | | |
| D51 | Blood, unit, 500ml | | | unit | | |
| D52 | Ferrous sulphate 200mg + folic acid 0,25mg | | | mg | | |
| | | | | | | |
| | 6. Lab tests | | | | | |
| D61 | Glycemy, PK50 | | | unit | | |
| D62 | GS/Rh 200tests, 10 ml | | | ml | | |
| D63 | Blood lancet, disposable | | | unit | | |
| D64 | Blood taking set with needle, disposable | | | unit | | |
| D65 | Ethanol 70% denaturated, 1 liter | | | ml | | |
| D66 | Gauze compresse 5*5cm (per 5 pieces) | | | unit | | |
| D67 | Hemoglobin test | | | unit | | |
| D68 | Hepapitis C rapid test | | | unit | | |
| D69 | Seringe 5ml with bypacked needle 21G | | | unit | | |
| D610 | Ultrasound procedure | | | unit | | |
| D611 | Urine collecting bag 2000ml, with tap and valve | | | unit | | |
| | | | | | | |
| | | | | | | |

| PART D : Patients suffering from shock | | | | | | |
|---|---|---|---|---|---|---|
| | 7. Anesthesics/sedatives | | | | | |
| D71 | Atropine 1mg/ml, 1ml for injection | | | mg | | |
| D72 | Diazepam, 10mg/2ml, for injection | | | mg | | |
| D73 | Fentanyl 0,1mg/2ml, for injection | | | mg | | |
| D74 | Halothane 250ml | | | ml | | |
| D75 | Ketamine 50mg/ml, 10ml for injection | | | mg | | |
| D76 | Lidocaïne HCl 1%, 20ml for injection | | | ml | | |
| D77 | Lidocaïne hydrochloride+ epinephrine | | | ml | | |
| D78 | Lidocaïne HCl 5%+dextrose 7,5%, 2ml for injection | | | ml | | |
| D79 | Thiopental sodium 1g, powder for injection | | | mg | | |
| D710 | Suxamethonium chloride 100mg/2ml for injection | | | mg | | |
| | | | | | | |
| | | | | | | |

| PART E : Patients suffering from cervix/vaginal laceration/perforation | | | | | | |
|---|---|---|---|---|---|---|
| N° | Name of input | Percent of **outpatients** suffering from cervix/vaginal laceration/perforation who received this input<br><br>0%=no women<br>100%=all women | Percent of **inpatients** suffering from cervix/vaginal laceration/perforation who received this input<br><br>0%=no women<br>100%=all women | Unit (**mg**=milligram, **mcg**=microgram, **ml**=milliliter **ui=**international unit **unit**=unit, piece… | Average number of basic units given per **outpatient** who receives this input | Average number of basic units given per **inpatient** who receives this input |
| E1 | Percent of women suffering from cervix/vaginal laceration/perforation who are treated as outpatients<br>0%=none treated as outpatients<br>100%=All treated as outpatients | % | % | | | |
| | 1. Anesthesics/sedatives | | | | | |
| E11 | Atropine 1mg/ml, 1ml for injection | | | mg | | |
| E12 | Diazepam, 10mg/2ml, for injection | | | mg | | |
| E13 | Fentanyl 0,1mg/2ml, for injection | | | mg | | |
| E14 | Halothane 250ml | | | ml | | |
| E15 | Ketamine 50mg/ml, 10ml for injection | | | mg | | |
| E16 | Lidocaïne HCl 1%, 20ml for injection | | | ml | | |
| E17 | Lidocaïne hydrochloride+ epinephrine | | | ml | | |
| E18 | Lidocaïne HCl 5%+dextrose 7,5%, 2ml for injection | | | ml | | |
| E19 | Thiopental sodium 1g, powder for injection | | | mg | | |
| E110 | Suxamethonium chloride 100mg/2ml for injection | | | mg | | |
| | | | | | | |
| | 2. Analgesics | | | | | |
| E21 | Acetylsalicylic acid, 100mg | | | mg | | |
| E22 | Diazepam, 10mg/2ml, for injection (valium) | | | mg | | |
| E23 | Diclofenac sodium, 75mg/3ml, for injection | | | mg | | |
| E24 | Diclofenac 100mg, rectal suppository | | | mg | | |
| E25 | Butilhisocin, 20g/ml, 1ml, for injection | | | mg | | |

| PART E : Patients suffering from cervix/vaginal laceration/perforation | | | | | | |
|---|---|---|---|---|---|---|
| E26 | Ibuprofen 400mg, film coated | | | mg | | |
| E27 | Metronidazole 500mg (metamizole) | | | mg | | |
| E28 | Paracetamol, tablets, 500mg | | | mg | | |
| E29 | Pethidine hydrochloride 50mg/ml, 1ml, for inj | | | mg | | |
| | | | | | | |
| | 3. Intravenous fluid replacement | | | | | |
| E31 | Ringer lactate, 1000ml, pvc bag | | | ml | | |
| E32 | Glucose isotonic 5%, intravenous solution 500ml, bag/bottle | | | ml | | |
| E33 | Glucose 5% + sodium chloride 0,9% IV, solution 500ml bag/bottle | | | ml | | |
| | | | | | | |
| | 4. Operating theater supplies | | | | | |
| E41 | Seringe 10ml, with bypacked needle 21G | | | ml | | |
| E42 | Seringe 60ml+catheter | | | ml | | |
| E43 | Gloves examination, latex, large,disposable | | | unit | | |
| E44 | Needle, disposable 21G*1.1/2 (0,8*38mm) | | | unit | | |
| E45 | Needle, disposable 18G*1.1/2 (1,2*38mm) | | | unit | | |
| E46 | Suture silk (0) 75cm with needle 30.0mm | | | cm | | |
| E47 | Cotton wool, 500g | | | mg | | |
| E48 | Gauze compresse 5*5cm, 12ply, non sterile | | | unit | | |
| E49 | Ethanol (alcool) 70%, denaturated, 1liter | | | ml | | |
| E410 | Povidone iodione solution 0,1 ; 200ml | | | ml | | |
| E411 | Tetanus vaccine 20doses/ unit | | | unit | | |
| | | | | | | |
| | | | | | | |
| | 5. Lab tests | | | | | |
| E51 | Glycemy, PK50 | | | unit | | |
| E52 | GS/Rh 200tests, 10 ml | | | ml | | |
| E53 | Blood lancet, disposable | | | unit | | |
| E54 | Blood taking set with needle, disposable | | | unit | | |
| E55 | Ethanol 70% denaturated, 1 liter | | | ml | | |
| E56 | Gauze compresse 5*5cm (per 5 pieces) | | | unit | | |

| | | | | | | |
|---|---|---|---|---|---|---|
| E57 | Hemoglobin test | | | Unit | | |
| E58 | Hepapitis C rapid test | | | Unit | | |
| E59 | Seringe 5ml with bypacked needle 21G | | | Unit | | |
| E510 | Ultrasound procedure | | | Unit | | |
| E511 | Urine collecting bag 2000ml, with tap and valve | | | Unit | | |
| | | | | | | |
| | 6. Antibiotics | | | | | |
| E61 | Amoxicillin 500mg | | | mg | | |
| E62 | Ampicillin, 1gram, powder for injection | | | mg | | |
| E63 | Ampicillin, tablets, 500mg | | | mg | | |
| E64 | Ceftriaxon, 1g, powder for injection | | | mg | | |
| E65 | Chloramphenicol, 1g, injection | | | mg | | |
| E66 | Ciprofloxacin, 500mg | | | mg | | |
| E67 | Doxycycline 100mg | | | mg | | |
| E68 | Gentamycin 80mg/2ml for injection | | | mg | | |
| E69 | Metronidazole injection 5mg/ml, 100ml | | | mg | | |
| E610 | Metronidazole 500mg vaginal | | | mg | | |
| | | | | | | |
| | 7. Others | | | | | |
| E71 | Tetanus vaccine 20 doses/ unit | | | unit | | |
| E72 | Ferrous sulphate 200mg + folic acid 0,25mg | | | mg | | |
| E73 | Dypron, Dipyrone 250mg/ml, 2ml, injection | | | mg | | |

| PART F : Patients suffering from uterus laceration/perforation | | | | | | |
|---|---|---|---|---|---|---|
| N° | Name of input | Percent of **outpatients** suffering from uterus laceration/perforation who received this input<br><br>0%=no women<br>100%=all women | Percent of **inpatients** suffering from uterus laceration/perforation who received this input<br><br>0%=no women<br>100%=all women | Unit (**mg**=milligram, **mcg**=microgram, **ml**=milliliter **ui=**international unit **unit**=unit, piece… | Average number of basic units given per **outpatient** who receives this input | Average number of basic units given per **inpatient** who receives this input |
| F1 | Percent of women suffering from uterus laceration/perforation who are treated as outpatients<br>0%=none treated as outpatients<br>100%=All treated as outpatients | % | % | | | |
| | 1. Anesthesics/sedatives | | | | | |
| F11 | Atropine 1mg/ml, 1ml for injection | | | mg | | |
| F12 | Diazepam, 10mg/2ml, for injection | | | mg | | |
| F13 | Fentanyl 0,1mg/2ml, for injection | | | mg | | |
| F14 | Halothane 250ml | | | ml | | |
| F15 | Ketamine 50mg/ml, 10ml for injection | | | mg | | |
| F16 | Lidocaïne HCl 1%, 20ml for injection | | | ml | | |
| F17 | Lidocaïne hydrochloride+ epinephrine | | | ml | | |
| F18 | Lidocaïne HCl 5%+dextrose 7,5%, 2ml for injection | | | ml | | |
| F19 | Thiopental sodium 1g, powder for injection | | | mg | | |
| F110 | Suxamethonium chloride 100mg/2ml for injection | | | mg | | |
| | | | | | | |
| | | | | | | |
| | 2. Analgesics | | | | | |
| F21 | Acetylsalicylic acid, 100mg | | | mg | | |
| F22 | Diazepam, 10mg/2ml, for injection (valium) | | | mg | | |
| F23 | Diclofenac sodium, 75mg/3ml, for injection | | | mg | | |
| F24 | Diclofenac 100mg, rectal suppository | | | mg | | |
| F25 | Butilhisocin, 20g/ml, 1ml, for injection | | | mg | | |

| PART F : Patients suffering from uterus laceration/perforation | | | | | | |
|---|---|---|---|---|---|---|
| F26 | Ibuprofen 400mg, film coated | | | mg | | |
| F27 | Metronidazole 500mg (metamizole) | | | mg | | |
| F28 | Paracetamol, tablets, 500mg | | | mg | | |
| F29 | Pethidine hydrochloride 50mg/ml, 1ml, for inj | | | mg | | |
| | | | | | | |
| | 3. Intravenous fluid replacement | | | | | |
| F31 | Ringer lactate, 1000ml, pvc bag | | | ml | | |
| F32 | Glucose isotonic 5%, intravenous solution 500ml, bag/bottle | | | ml | | |
| F33 | Glucose 5% + sodium chloride 0,9% IV, solution 500ml bag/bottle | | | ml | | |
| F34 | Polygeline 4%, 500ml (gelofusine) | | | ml | | |
| F35 | Sodium chloride 0,9%, 1000ml bag | | | ml | | |
| | | | | | | |
| | 4. Operating theater supplies | | | | | |
| F41 | Seringe 10ml, with needle 21G*1,5 inch | | | ml | | |
| F42 | Seringe 60ml+catheter, disposable | | | ml | | |
| F43 | Gloves examination, latex, large,disposable | | | unit | | |
| F44 | Needle, disposable 21G*1.1/2 (0,8*38mm) | | | unit | | |
| F45 | Needle, disposable 18G*1.1/2 (1,2*38mm) | | | unit | | |
| F46 | Suture silk (0) 75cm with needle 30.0mm | | | cm | | |
| F47 | Cotton, 500g | | | mg | | |
| F48 | Suture silk vicryl (3/0) 2*70cm | | | unit | | |
| F49 | Suture silk vicryl (3/0), 70cm, 17,5mm | | | cm | | |
| F410 | Suture needles assorted sizes | | | unit | | |
| F411 | Gauze compresse 5*5cm, 12ply, non sterile | | | unit | | |
| F412 | Ethanol (alcool) 70%, denaturated, 1liter | | | ml | | |
| F413 | Povidone iodine solution 0,1 ; 200ml | | | ml | | |
| F414 | Epinephrine (adrenaline) 1mg/ml, 1ml, injection | | | mg | | |
| F415 | Water for injection, 10ml | | | ml | | |
| F416 | Blood, ( one unit=500ml) | | | unit | | |
| F417 | Oxygen | | | ml | | |
| F418 | Tetanus vaccine 20 doses ( unit) | | | unit | | |

| PART F : Patients suffering from uterus laceration/perforation | | | | | | |
|---|---|---|---|---|---|---|
| F419 | Cetrimide 15% + chlorhexidine gluconate 1,5%, 1l | | | ml | | |
| | | | | | | |
| | 5. Supplies for laparotomy | | | | | |
| F51 | Gloves examination, latex, large, disposable | | | Unit | | |
| F52 | Sanitary pad | | | unit | | |
| F53 | Gauze compresses 100cm, 12 ply, sterile per piece | | | cm | | |
| F54 | Cotton swab | | | Unit | | |
| F55 | Suture silk 75cm with needle 30mm | | | Unit | | |
| F56 | Drapes in strong cotton 1,5$m^2$ | | | unit | | |
| | | | | | | |
| | | | | | | |
| | 6. Lab tests | | | | | |
| F61 | Glycémy, PK50 | | | Unit | | |
| F62 | GS/Rh 200tests, 10 ml | | | ml | | |
| F63 | Blood lancet, disposable | | | Unit | | |
| F64 | Blood taking set with needle, disposable | | | Unit | | |
| F65 | Ethanol 70% denaturated, 1 liter | | | ml | | |
| F66 | Gauze compresse 5*5cm (per 5 pieces) | | | Unit | | |
| F67 | Hemoglobin test | | | Unit | | |
| F68 | Hepapitis C rapid test | | | Unit | | |
| F69 | Seringe 5ml with bypacked needle 21G | | | Unit | | |
| F610 | Ultrasound procedure | | | unit | | |
| F611 | Urine collecting bag 2000ml, with tap and valve | | | unit | | |
| | | | | | | |
| | 7. Antibiotics | | | | | |
| E61 | Amoxicillin 500mg | | | mg | | |
| E62 | Ampicillin, 1gram, powder for injection | | | mg | | |
| E63 | Ampicillin, tablets, 500mg | | | mg | | |
| E64 | Ceftriaxon, 1g, powder for injection | | | mg | | |

| | | | | | | |
|---|---|---|---|---|---|---|
| E65 | Chloramphenicol, 1g, injection | | | mg | | |
| E66 | Ciprofloxacin, 500mg | | | mg | | |
| E67 | Doxycycline 100mg | | | mg | | |
| E68 | Gentamycine 80mg/2ml for injection | | | mg | | |
| E69 | Metronidazole injection 5mg/ml, 100ml | | | mg | | |
| E610 | Metronidazole 500mg vaginal | | | mg | | |
| | | | | | | |
| | 8. Others | | | | | |
| E71 | Tetanus vaccine 20 doses/ unit | | | unit | | |
| E72 | Ferrous sulphate 200mg + folic acid 0,25mg | | | mg | | |
| E73 | Dypron, Dipyrone 250mg/ml, 2ml, injection | | | mg | | |
