# Supplementary material for: Estimating the costs for the treatment of abortion complications in two public referral hospitals: a cross-sectional study in Ouagadougou, Burkina Faso
Source: BMC Health Serv Res. 2016 Oct 7;16:559. doi: 10.1186/s12913-016-1822-7 (PMC5055714; doi:10.1186/s12913-016-1822-7)
Supplement: Additional file 2: — This questionnaire was designed to collect indirect costs borne by hospital facilities in treating patients. (PDF 327 kb) [file 12913_2016_1822_MOESM2_ESM.pdf]

ABORTION CARE COSTS TO HOSPITALS

This questionnaire must be completed by interviewing keys staff of the hospital and maternity ward.

Section A : Identification of hospital

A1. Name of hospital?

CHU-YO ☐ (1) CMA 30 ☐ (2)

A2. Date of interview: |_|_|_|_|_|_|

A3. Name and surname of investigator……………………………………………………………

Section B : Identification of interviewed persons

B1. Name and surname of the first interviewed person……………………………………………..

B2. Title of the first interviewed person ……………………………………………………….

B3. Telephone of the first interviewed person …………………………………………………

B4. Name and surname of the second interviewed person……………………………………….

B5. Title of the second interviewed person ……………………………………………………

B6. Telephone of the second interviewed person …………………………………………..

B7. Name and surname of the third interviewed person……………………………………

B8. Title of the third interviewed person ………………………………………………………

B9. Telephone of the third interviewed person ……………………………………………..

<u>Section C</u> : Abortion and complications

C1. Do you have any data on abortion taken in charge by your structure?

Yes ☐ (1) No ☐ (2)→**Go to C3**

C2. How many women were taken in charge following abortion last year i.e in 2010?

☐☐☐☐☐ (Don't know=97979)

C3. Among those women who were taken in charge following abortion, how many were taken in charge for complications? ☐☐☐☐☐ (Don't know=97979)

For this survey, it is important to have a good estimation of the distribution of the cases of complications of abortion. For the following questions, you are asked to think of <u>100 women</u> who consult following complication of abortion

C4. How many women out of <u>100</u> taken in charge following abortion complications have been treated for :

Incomplete abortion ☐☐ (Don't know=97)

Sepsis/infection ☐☐ (Don't know=97)

Haemorrhage ☐☐ (Don't know=97)

Shock ☐☐ (Don't know=97)

Vagina or cervix laceration/perforation ☐☐ (Don't know=97)

Uterus laceration/perforation ☐☐ (Don't know=97)

Other complications ☐☐ (Don't know=97)

<u>Section D</u> : Personnel costs

You are asked to think of only <u>10 women</u> admitted in your structure following incomplete abortion with just only one complication.

D1. Out of 10 women admitted following **incomplete abortion**, how many of them will be hospitalized? ☐☐ (Don't know=97)

D2. Of those hospitalized following **incomplete abortion,** what is the average days of hospitalization? ☐☐ (Don't know=97)

D3. Out of <u>10 women</u> taken in charge for **<u>incomplete abortion</u>** in your structure, how many of them are treated by each of the following medical personnel and on the average how long does each of the personnel give over to taking charge of the woman for incomplete abortion from admittance to discharge?

N°	Category of medical staff	Has she been taken in charge by the agent Yes=1 ; No=0	Number of women out of 10 seen by each of the member staff	Time (mn) spent by each member staff in taking charge of an incomplete abortion
1	Gynaecologist			
2	Anesthesist/ assistant anesthesist			
3	Operatar/ assistant operator			
4	Clinician			
5	Midwife/ maieuticist			
6	Assistant midwife			
7	Nurse			
8	Laboratory technician			
9	Echographer			
10	Pharmacist			
11	Medecine distributor			
12	Counsellor			
13	Other1 (specify……………………			
14	Other2 (specify……………………			
15	Other3 (specify……………………			

D4. Out of 10 women admitted following **sepsis/infection**, how many of them will be hospitalized? ☐☐ (Don't know=97)

D5. Of those hospitalized following **sepsis/infection,** what is the average days of hospitalization? ☐☐ (Don't know=97)

D6. Out of 10 women taken in charge for **sepsis/infection** in your structure, how many of them are treated by each of the following medical personnel and on the average how long does each of the personnel give over to taking charge of the woman for incomplete abortion from admittance to discharge?

N°	Category of medical staff	Has she been taken in charge by the agent Yes=1 ; No=0	Number of women out of 10 seen by each of the member staff	Time (mn) spent by each member staff in taking charge of an incomplete abortion
1	Gynaecologist			
2	Anesthesist/ assistant anesthesist			
3	Operatar/ assistant operator			
4	Clinician			
5	Midwife/ maieuticist			
6	Assistant midwife			
7	Nurse			
8	Laboratory technician			
9	Echographer			
10	Pharmacist			
11	Medecine distributor			
12	Counsellor			
13	Other1 (specify……………………			
14	Other2 (specify……………………			
15	Other3 (specify……………………			

D7. Out of 10 women admitted following **haemorrhage**, how many of them will be hospitalized? ☐☐ (Don't know=97)

D8. Of those hospitalized following **haemorrhage,** what is the average days of hospitalization? ☐☐ (Don't know=97)

D9. Out of 10 women taken in charge for **haemorrhage** in your structure, how many of them are treated by each of the following medical personnel and on the average how long does each of the personnel give over to taking charge of the woman for incomplete abortion from admittance to discharge?

N°	Category of medical staff	Has she been taken in charge by the agent Yes=1 ; No=0	Number of women out of 10 seen by each of the member staff	Time (mn) spent by each member staff in taking charge of an incomplete abortion
1	Gynaecologist			
2	Anesthesist/ assistant anesthesist			

3	Operatar/ assistant operator			
4	Clinician			
5	Midwife/ maieuticist			
6	Assistant midwife			
7	Nurse			
8	Laboratory technician			
9	Echographer			
10	Pharmacist			
11	Medecine distributor			
12	Counsellor			
13	Other1 (specify……………………			
14	Other2 (specify……………………			
15	Other3 (specify……………………			

D10. Out of 10 women admitted following **shock**, how many of them will be hospitalized?

☐☐ (Don't know=97)

D11. Of those hospitalized following **shock,** what is the average days of hospitalization? ☐☐ (Don't know=97)

D12. Out of 10 women taken in charge for **shock** in your structure, how many of them are treated by each of the following medical personnel and on the average how long does each of the personnel give over to taking charge of the woman for incomplete abortion from admittance to discharge?

N°	Category of medical staff	Has she been taken in charge by the agent Yes=1 ; No=0	Number of women out of 10 seen by each of the member staff	Time (mn) spent by each member staff in taking charge of an incomplete abortion
1	Gynaecologist			
2	Anesthesist/ assistant anesthesist			
3	Operator/ assistant operator			
4	Clinician			
5	Midwife/ maieuticist			
6	Assistant midwife			
7	Nurse			
8	Laboratory technician			
9	Echographer			
10	Pharmacist			
11	Medecine distributor			
12	Counsellor			
13	Other1 (specify……………………			
14	Other2 (specify……………………			
15	Other3 (specify……………………			

D13. Out of 10 women admitted following **vagina or cervix laceration/perforation**, how many of them will be hospitalized? ☐☐ (Don't know=97)

D14. Of those hospitalized following **vagina or cervix laceration/perforation,** what is the average days of hospitalization? ☐☐ (Don't know=97)

D15. Out of <u>10 women</u> taken in charge for **vagina or cervix laceration/perforation** in your structure, how many of them are treated by each of the following medical personnel and on the average how long does each of the personnel give over to taking charge of the woman for incomplete abortion from admittance to discharge?

N°	Category of medical staff	Has she been taken in charge by the agent Yes=1 ; No=0	Number of women out of 10 seen by each of the member staff	Time (mn) spent by each member staff in taking charge of an incomplete abortion
1	Gynaecologist			
2	Anesthesist/ assistant anesthesist			
3	Operatar/ assistant operator			
4	Clinician			
5	Midwife/ maieuticist			
6	Assistant midwife			
7	Nurse			
8	Laboratory technician			
9	Echographer			
10	Pharmacist			
11	Medecine distributor			
12	Counsellor			
13	Other1 (specify……………………			
14	Other2 (specify……………………			
15	Other3 (specify……………………			

D16. Out of 10 women admitted following **uterus laceration/perforation**, how many of them will be hospitalized? ☐☐ (Don't know=97)

D17. Of those hospitalized following **uterus laceration/perforation,** what is the average days of hospitalization? ☐☐ (Don't know=97)

D18. Out of <u>10 women</u> taken in charge for **uterus laceration/perforation** in your structure, how many of them are treated by each of the following medical personnel and on the average how long does each of the personnel give over to taking charge of the woman for incomplete abortion from admittance to discharge?

N°	Category of medical staff	Has she been taken in charge by the agent Yes=1 ; No=0	Number of women out of 10 seen by each of the member staff	Time (mn) spent by each member staff in taking charge of an incomplete abortion
1	Gynaecologist			
2	Anesthesist/ assistant anesthesist			
3	Operatar/ assistant operator			
4	Clinician			
5	Midwife/ maieuticist			
6	Assistant midwife			
7	Nurse			
8	Laboratory technician			
9	Echographer			

10	Pharmacist			
11	Medecine distributor			
12	Counsellor			
13	Other1 (specify……………………			
14	Other2 (specify……………………			
15	Other3 (specify……………………			

D19. Do patients pay hospitalization fees in your structure?

Yes ☐ (1) No ☐ (2)→**Go to E1**

D20. How much do they pay for hospitalization? ☐☐☐☐☐☐ (Don't know =979797)

<u>Section E</u>: Staff costs

E1. On average how many women are taken in charge in your structure each year

☐☐☐☐☐☐

E2. Out of those patients taken in charge, how many of them have been taken in charge in the maternity service (including contacts by new-born ones and other obstetrical complications)

☐☐☐☐☐☐

Now, you are asked to give estimate costs of all investments ; of course, it is difficult to have a precise estimation of investment costs. That's why as an expert, you are asked to give us the best possible estimation of those costs.

E3. How much do you think it's necessary to build and equip a structure such as yours? ☐

E4. How long do you think such structure can last (in years)? ☐

E5. How much do you estimate the average gross wage of each category of staff in your structure?

N°	Category of medical staff	Average gross wage in CFA of each category of medical staff
1	Gynaecologist	
2	Anesthesist/ assistant anesthesist	
3	Operator/ assistant operator	
4	Clinician	
5	Midwife/ maieuticist	
6	Assistant midwife	
7	Nurse	
8	Laboratory technician	
9	Echographer	
10	Pharmacist	
11	Medecine distributor	
12	Counsellor	
13	Other1 (specify……………………	
14	Other2 (specify……………………	
15	Other3 (specify……………………	

*Now, youare asked to give an estimation of the costs of overheads. For sure, it is difficult to have a precise estimation of the costs of overheads. That's why as an expert, you are asked to give us the best possible estimation of the costs for **non medical staff**.*

E6. Can you tell me, how many people from each category for the non medical staff work in your structure and indicate their average gross wage?

N°	Category of non medical staff	Number of staff by category of work	Average gross wage in CFA by category
1	Guardians/caretakers		
2	Cleaners		
3	Drivers		
4	Ward erderly		
5	Stretch-bearers		
6	Cooks		
7	Laundry workers		
8	Other1………………………………		
9	Other 2………………………………		
10	Other 3………………………………		
11	Other 4………………………………		
12	Other 5………………………………		

E7. Can you give and estimation of the following overheads in your structure?

N°	Kind and type of overheads	Costs in CFA
1	Maintenance of buildings	
2	Total costs of services (water, electricity, gas, telephone…)	
3	Maintenance of rolling stocks	
4	Transport and travels (fuel, meals, lodging…)	
5	Audio-visual equipment	
6	Background papers of the staff	
7	Printed forms and related materials	
8	Other1………………………………	
9	Other 2………………………………	
10	Other 3………………………………	
11	Other 4………………………………	
12	Other 5………………………………	

Thank you for your answers.
